# The combination of gene hyperamplification and PD-L1 expression as a biomarker for the clinical benefit of tislelizumab in gastric/gastroesophageal junction adenocarcinoma

**DOI:** 10.1007/s10120-022-01308-7

**Published:** 2022-07-02

**Authors:** Zhihao Lu, Silu Yang, Xuerui Luo, Yang Shi, Jong-Seok Lee, Sanjeev Deva, Tianshu Liu, Yee Chao, Yun Zhang, Ruiqi Huang, Yaling Xu, Zhirong Shen, Lin Shen

**Affiliations:** 1 Key Laboratory of Carcinogenesis and Translational Research (Ministry of Education/Beijing), Peking University Cancer Hospital and Institute, Beijing, China; 2 BeiGene (Beijing) Co., Ltd., Beijing, China; 3 BeiGene (Shanghai) Co., Ltd., Shanghai, China; 4 Seoul National University Bundang Hospital, Seoul, South Korea; 5 Auckland City Hospital, Auckland, New Zealand; 6 Zhongshan Hospital Fudan University, Shanghai, China; 7 Taipei Veterans General Hospital, Taipei City, Taiwan

**Keywords:** Immuno-oncology, Gastroesophageal adenocarcinoma, PD-(L)1 inhibitor, PD-L1, Hyperamplification

## Abstract

**Background:**

In solid tumor Phase 1/2 trials (NCT02407990; NCT04068519), tislelizumab demonstrated clinical benefit, including in advanced gastroesophageal adenocarcinoma (GEA). However, the majority of patients with GEA did not respond, highlighting the need to understand mechanisms of resistance and identify predictive biomarkers for response.

**Methods:**

All tislelizumab-treated patients with GEA from the Phase 1/2 trials were included (*N* = 105). Programmed death-ligand 1 (PD-L1) expression (Tumor Area Positivity [TAP] ≥ 5%), interferon gamma (IFN*γ*)-related gene signature, gene expression profile, tumor mutational burden (TMB), and gene hyperamplification (HA) were analyzed for correlation with tislelizumab.

**Results:**

A moderate association was observed between PD-L1 TAP ≥ 5%, IFN*γ* gene signature, TMB-high and efficacy. A potential correlation between hyperamplification (HA +) and worse outcomes with programmed cell death protein 1 (PD-1) inhibition was identified. Hyperamplified genes were mainly enriched in cancer progression pathways, including cell cycle and RTK-RAS-PI3K pathways. Joint PD-L1 TAP ≥ 5% and lack of hyperamplification showed the most favorable benefit with an objective response rate of 29.4%, and median progression-free survival and overall survival of 4.1 and 14.7 months, respectively. Tumors with TAP ≥ 5% and HA − had inflamed immune signatures with increased immune cell infiltration, enhanced anti-tumor cytotoxic activity and antigen presentation signatures. Findings were validated in two independent gastric and gastrointestinal cancer cohorts treated with immune checkpoint inhibitors.

**Conclusions:**

In GEA, PD-L1 positivity, IFN*γ*-related gene signature and TMB-high status were positively associated with tislelizumab clinical benefit, whereas HA was associated with worse clinical outcomes. Combining PD-L1 positivity and HA − may help identify patients more likely to benefit from PD-1 blockade.

**Supplementary Information:**

The online version contains supplementary material available at 10.1007/s10120-022-01308-7.

## Introduction

Gastroesophageal adenocarcinoma (GEA) is a major cause of cancer-related mortality worldwide that is usually diagnosed at advanced or metastatic stages, with limited treatment options and a poor prognosis [1, 2]. Programmed cell death protein 1 (PD-1) inhibitors have demonstrated antitumor activity and survival benefit in patients with GEA. However, only a limited number of patients respond to treatment, with objective response rates (ORR) typically ≤ 16% in clinical trials, highlighting an urgent need to understand mechanisms of resistance and identify predictive biomarkers for response [3–8].

Several studies have sought to identify predictive biomarkers for PD-1 inhibitor response in GEA, with limited success. Programmed death-ligand 1 (PD-L1) expression status is one of the most widely used biomarkers in immunotherapy. In the Phase 2 KEYNOTE-059 study of pembrolizumab in recurrent or metastatic gastric or gastroesophageal junction (G/GEJ) adenocarcinoma, patients with a PD-L1 positive status (combined positive score [CPS] ≥ 1) had a higher ORR compared with patients with a PD-L1 negative status (CPS < 1) (15.5 versus 6.4%, respectively) [7]. Based on this study, the PD-L1 immunohistochemistry (IHC) 22C3 PharmDx test (Dako, Agilent, Santa Clara, CA, USA) was approved by the US Food and Drug Administration (FDA) concurrently with pembrolizumab for patients with gastric cancer (GC). However, in the more recent Phase 3 KEYNOTE-061 and -062 studies, pembrolizumab failed to demonstrate statistically superior overall survival (OS) compared with chemotherapy in patients with G/GEJ and a CPS ≥ 1 [5, 8].

Beyond PD-L1 expression, a 6-gene interferon gamma (IFNγ)-related signature based on gene expression profiling was tested in a small population of pembrolizumab-treated patients with G/GEJ adenocarcinoma (*N* = 33) [9]. Tumors that responded to pembrolizumab showed a numerically higher signature score [9]. To validate this observation, a refined 18-gene *T* cell-inflamed gene expression profiling score was investigated in a larger independent cohort (*N* = 144) and revealed a higher combined score in responders than in non-responders (*P* = 0.010) and was associated with improved progression-free survival (PFS) (*P* = 0.002) [7].

Other than immune-related extrinsic biomarkers, various tumor intrinsic biomarkers have also been explored. In G/GEJ cancer, a subgroup analysis of KEYNOTE-059, -061 and -062 identified a small group of patients (*n* = 84) with microsatellite instability high (MSI-H) tumors. These patients demonstrated an improved OS and clinical response to pembrolizumab compared with paclitaxel [10]. Similarly, exploratory analyses of KEYNOTE-061 and -062 revealed that tumor mutational burden-high (TMB-high) status was strongly associated with improved clinical outcomes with pembrolizumab but not chemotherapy in both the first- and second-line treatment settings [11–13]. More recently, emerging evidence has pointed to the predictive role of somatic copy-number alternation (CNA) in patients treated with checkpoint inhibitors [14–16]. A study in a gastrointestinal (GI) cohort treated with checkpoint inhibitors revealed lower CNA burden was associated with improved clinical benefit and survival [17].

Tislelizumab is a humanized monoclonal antibody with high affinity and specificity for PD-1 that was specifically designed to minimize Fcɣ receptor binding on macrophages to abrogate antibody-dependent phagocytosis, a potential mechanism of* T* cell clearance and resistance to anti-PD-1 therapy [18–20]. Tislelizumab monotherapy demonstrated an acceptable safety and tolerability profile, anti-tumor activity, and durable responses in heavily pre-treated patients with advanced solid tumors in a Phase 1a/1b study (NCT02407990) and in Chinese patients with advanced or metastatic solid tumors in a Phase 1/2 study (NCT04068519) [21, 22]. Here, we report the relationship between biomarkers and clinical efficacy in a cohort of patients with GEA, treated with tislelizumab monotherapy, from these two trials.

## Patients and methods

### Study design and patient population

The study designs of NCT02407990 and NCT04068519 have been described previously [21, 22]. Eligible patients were aged ≥ 18 years with previously treated histologically or cytologically confirmed advanced or metastatic solid tumors with measurable disease (defined by Response Evaluation Criteria in Solid Tumors version 1.1 [RECIST v.1.1]) [23], and an Eastern Cooperative Oncology Group (ECOG) performance status of 0 or 1. Key exclusion criteria included prior therapy with agents targeting PD-1 or PD-L1 and symptomatic brain metastases.

In this post-hoc pooled analysis, data from 105 tislelizumab-treated patients diagnosed with GEA, including G/GEJ adenocarcinoma (*n* = 78) and esophageal adenocarcinoma (EAC) (*n* = 27), were retrospectively collected.

### Clinical endpoints

The following clinical outcomes were evaluated in the GEA cohort: investigator-assessed ORR, disease control rate (DCR), PFS and OS. Tumor response was assessed by computed tomography imaging or other radiological assessments at screening, every 8 or 9 weeks for the first 12 months, and every 12 weeks thereafter [21, 22]. ORR was defined as the proportion of patients who had a complete response (CR) or a partial response (PR), while DCR was defined as the proportion of patients who achieved CR, PR, or stable disease (SD) per RECIST v.1.1 criteria [23]. Non-responders included patients who had SD or progressive disease (PD), or tumors that were not evaluable (NE).

### Biomarker assessments

All genetic alteration, gene expression and immunohistology analysis in this cohort were performed on formalin-fixed paraffin-embedded (FFPE) tumor tissue samples collected at the screening stage.

PD-L1 expression was retrospectively assessed using VENTANA PD-L1 (SP263) IHC assay (Ventana Medical Systems, Oro Valley, AZ, USA). PD-L1 expression levels were scored by Tumor Area Positivity (TAP) score, which is a validated algorithm that uses visual estimation of PD-L1 expression on tumor and immune cells. TAP ≥ 5% stained by the VENTANA PD-L1 (SP263) assay was determined as the optimal cut-off based on receiver operating characteristic (ROC) results in tumor samples of G/GEJ adenocarcinoma [24].

CD8 was retrospectively assessed using the VENTANA anti-CD8 (SP57) assay in a subset of patients from NCT02407990. For qualitative detection of CD8 in neoplastic tissues, the whole immunostained slides were scanned at 40 × magnification by VENTANA iScan HT (Roche). Image analysis was performed using the HALO software package (Indica Labs, Albuquerque, NM, USA). Tumor/stroma classification and nuclei segmentation were reviewed by two blinded pathologists using the Indica-Multiple IHC v3.1.4 module. The density of the CD8 immunostained *T* cells was determined by dividing the number of CD8 *T* cells (including cells stained with 3 + , 2 + , and 1 + intensity) by the examined area in mm^2^.

Gene expression data were generated using HTG EdgeSeq Precision Immuno-Oncology Panel (HTG Molecular Diagnostics, Inc., Tucson, AZ, USA) on baseline tumor samples, per the manufacturer’s instructions. The library was sequenced on the Illumina Nextseq 500 platform (Illumina, Inc., San Diego, CA, USA) and data were processed by HTG EdgeSeq parser software. Read count was normalized by library size to get counts-per-million (CPM) reads, which was then log transformed. The pan-tumor 18-gene* T* cell inflamed signature (TIS) score (*CCL5, CD27, CD274 [PD-L1], CD276 [B7-H3], CD8A, CMKLR1, CXCL9, CXCR6, HLA-DQA1, HLA-DRB1, HLA-E, IDO1, LAG3, NKG7, PDCD1LG2 [PD-L2], PSMB10, STAT1, TIGIT*) [9], 6-gene IFNγ-related signature (*IFNγ, CXCL9, CXCL10, IDO1, STAT1, HLA-DRA*) and other gene expression signatures were calculated using Gene Set Variation Analysis (GSVA) [25].

Genetic alteration profiling and TMB were assessed using FoundationOne CDx Assay (F1CDx; Foundation Medicine, Cambridge, MA, USA) [26] using tumor tissues from study NCT02407990 [21] and BurningRock OncoScreen Plus 520 panel (BR520; OncoScreen Plus, Burning Rock Biotech, China) [27] for study NCT04068519 [22]. To assess the appropriateness of TMB analysis using the combined results from these two different assays, sequencing on a small batch of samples from advanced solid tumors (*N* = 54) was performed, which demonstrated that the TMB levels derived from the two assays were highly correlated (Spearman correlation = 0.804; Supplementary Fig. 1). TMB level was defined as low (TMB-low; < 8 mut/Mb), or high (TMB-high; ≥ 8 mut/Mb), based on the threshold recommended in ROC curves between responders and non-responders. A tumor was defined as having hyperamplification (HA +) if any gene in the common set of genes shared by both F1CDx and BR520 panels was amplified with copy number (CN) > 5. This threshold was derived from the criteria used within the F1CDx assay and has been shown to have high concordance (accuracy > 95%) with results obtained from other techniques, such as fluorescence in situ hybridization and immunohistochemistry [28].

### Independent cohort analyses

Three independent cohorts were used for validation. First, a study of Korean patients with GC treated with pembrolizumab was used as an independent GC validation cohort [29]. Raw sequencing data (45 tumors profiled by RNA-sequencing [RNA-seq] and 55 tumors profiled by whole exome sequencing [WES]) was retrieved from the European Nucleotide Archive (ENA) database under accession PRJEB25780 (https://www.ebi.ac.uk/ena/browser/view/PRJEB25780) [30]. Reads from RNA-seq were aligned to the human genome (hg19) using Spliced Transcripts Alignment to a Reference (STAR) [31]. RNA-Seq by Expectation Maximization (RSEM) pipeline was subsequently applied to establish the fragments per kilobase per million mapped reads (FPKM) of each gene, which was normalized for downstream analysis [32]. For WES reads, raw reads were mapped to hg19, duplicated reads were removed and single-nucleotide variants and indels were identified using Sentieon^®^ (Sentieon. Inc, San Jose, CA, USA) with recommended parameters [33]. CNA were detected using CNVkit [34]. The definition of HA + was identical to that in the tislelizumab-treated cohort. PD-L1 expression level was determined according to CPS from Dako PD-L1 IHC 22C3 pharmDx assay (Agilent Technologies). Specimens with PD-L1 expression CPS ≥ 1 were considered as PD-L1 positive [29].

Secondly, a previously reported study in Chinese patients with GI cancer (including GC, esophageal cancer and colorectal cancer) treated with immune checkpoint inhibitors (anti-PD-[L]1 and/or anti-CTLA-4 antibodies) was used as an independent GI cancer validation cohort [17, 35]. A total of 82 FFPE tumor tissues from this GI cohort were profiled with WES, as described in the original manuscript [17, 35]. CNAs identified in the original study were used to define HA + based on the same standards in our tislelizumab-treated cohort.

Thirdly, data from 150 patients in the Cancer Genome Atlas (TCGA; https://www.cancer.gov/tcga) stomach adenocarcinoma (STAD) cohort reported to have received chemotherapy were used for a further validation cohort. All genomic data (mutation, I, gene expression) and clinical data of the STAD cohort were retrieved from the Genomic Data Commons data portal (https://portal.gdc.cancer.gov/). Gene expression levels were calculated using RNA-seq data. Due to the absence of PD-L1 assay in the TCGA database, we use CD274 RNA expression to represent PD-L1 expression. CD274^high^ and CD274^low^ were determined according to the median value of CD274 RNA level. Furthermore, the criteria used to define HA + in this cohort differed slightly from the tislelizumab-treated patients owing to the use of criteria defined by the TCGA work group using GISTIC2.0. Using the same common set of genes (F1CDx and BR520 panels), each gene was assigned to one of five categories: deep deletion, deletion, normal, shallow amplification, and focal amplification. Focal amplification represented the highest amplification level, and most closely matched the definition of HA + in the tislelizumab-treated patients, therefore, was used as the definition for HA + .

### Statistical analysis

The data used in this analysis were final at the database lock date of August 26, 2020, for trial NCT02407990 and May 31, 2020, for NCT04068519. Statistical analyses and visualizations were performed using R 34.0.2.6.2 or SAS 9.4. Descriptive analyses for demographic and baseline information was performed for the overall biomarker analysis cohort and by study. Investigator-assessed ORR (determined by RECIST v1.1) was summarized for each biomarker-defined subgroup, and the 95% confidence interval (CI) of the ORR was constructed. Fisher’s exact test was used to compare ORR between biomarker-defined subgroups. For comparisons among two biomarker-defined subgroups, the Bonferroni method was used for multiplicity adjustment. Other binary outcomes, such as DCR, were similarly analyzed. Similar analysis for binary outcomes was followed for independent validation cohorts. For time-to-event outcomes, including PFS and OS, median survival was estimated using the Kaplan–Meier method. Log-rank test was used to compare survival curves between/among biomarker(s)-defined subgroups. *P* values were adjusted if there were multiple comparisons. Further exploration of the association of HA with other markers in clinical outcomes using model-based methods was also conducted. For binary response outcome, logistic regression was applied. For PFS and OS, Cox proportional hazard model was used. The corresponding point estimates (odds ratio and hazard ratio) and associated 95% CI were provided. All statistical analyses are post-hoc and exploratory, and *P* values are descriptive only.

## Results

### Baseline characteristics and clinical outcomes

A total of 105 patients with GEA were enrolled from studies NCT02407990 (*n* = 81) and NCT04068519 (*n* = 24) between September 2015 and May 2018 and received tislelizumab monotherapy. Across these two studies, baseline characteristics were generally similar except for race and primary tumor site, due to differences between the study protocols. Half of patients (50.5%) had received ≥ 3 lines of prior systemic anticancer treatment and most patients (95.2%) had Stage IV disease at baseline. At a median follow-up of 32.8 months, ORR was 11.4% (95% CI 6.1–19.1), and mPFS and mOS were 2.0 months (95% CI 1.9–2.1) and 5.7 months (95% CI 4.3–8.6), respectively (Supplementary Table 1).

### Association of biomarkers with clinical outcomes

A total of 92 patients (87.6%) had evaluable PD-L1 expression, 74 (70.5%) had evaluable genomic profiles, 63 (60.0%) had evaluable TMB status, and 80 (76.2%) had evaluable GEP including 6-gene IFN*γ* and TIS score. The baseline characteristics and clinical outcomes of the biomarker-evaluable populations (BEP) were generally comparable to the overall population (Supplementary Table 2).

**Fig. 1:**
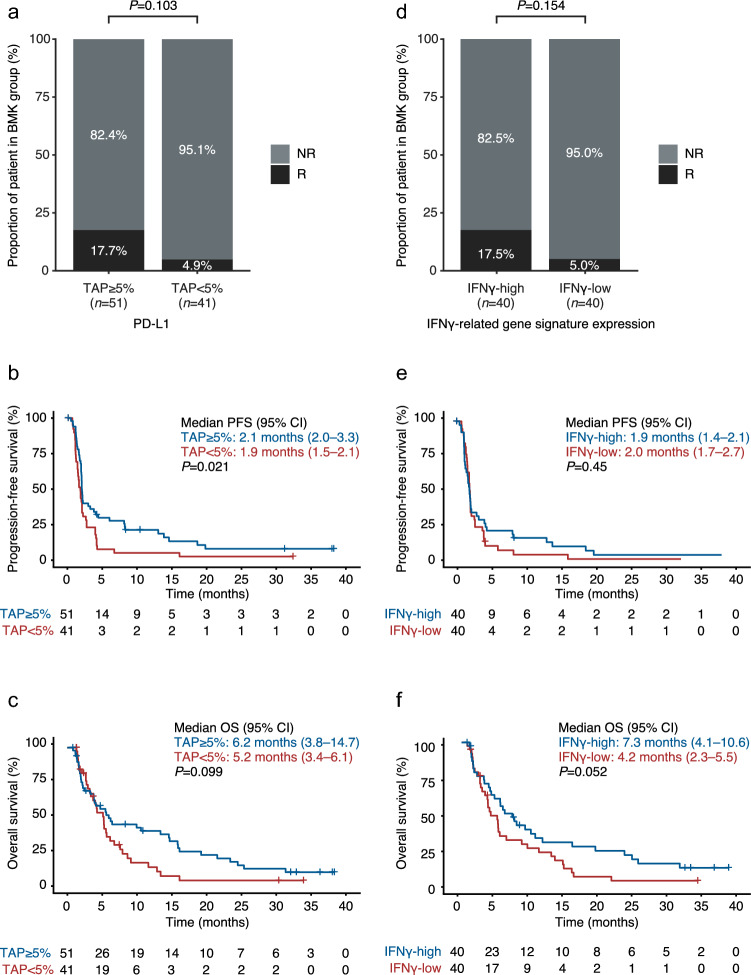
Association of PD-L1 TAP and IFN*γ*-related gene signature expression with clinical outcome of tislelizumab monotherapy in the tislelizumab-treated GEA cohort. **a** Objective response rates according to PD-L1 status, **b** Kaplan–Meier plot for PFS according to PD-L1 status, **c** Kaplan–Meier plot for OS according to PD-L1 status, **d** objective response rates according to IFN*γ*-related gene signature expression, **e** Kaplan–Meier plot for PFS according to IFN*γ*-related gene signature expression, **f** Kaplan–Meier plot for OS according to IFN*γ*-related gene signature expression. *BMK* biomarker, *CI* confidence interval, *GEA* gastroesophageal adenocarcinoma, *IFNγ* interferon gamma, *mOS* median OS, *mPFS* median PFS, *NR* non-responder, *OS* overall survival, *PD-L1* programmed death-ligand 1, *PFS* progression-free survival, *R* responder, *TAP* Tumor Area Positivity

The association between PD-L1 expression and tislelizumab clinical efficacy was evaluated first. Higher ORR and longer survival were observed in patients with TAP ≥ 5% (55.4% of the BEP) versus TAP < 5% (ORR: 17.7% versus 4.9% [*P* = 0.103]; mPFS: 2.1 versus 1.9 months [*P* = 0.021]; mOS: 6.2 versus 5.2 months [*P* = 0.099], respectively) (Fig. 1a–c).

Examination of the 6-gene IFN*γ* score and clinical efficacy showed a trend toward correlation in tumors from responders versus non-responders. With a median IFN*γ* signature score as cutoff, ORR was enriched in patients with IFN*γ*-high versus IFN*γ*-low (ORR: 17.5% versus 5.0% [*P* = 0.154], respectively), and there was a trend for favorable OS in patients with IFN*γ*-high versus IFN*γ*-low (mOS: 7.3 versus 4.2 months [*P* = 0.052], respectively), but PFS was similar in the two groups (mPFS: 1.9 versus 2.0 months [*P* = 0.45], respectively) (Fig. 1d–f). Additionally, a consistent trend was identified between TIS score status and tislelizumab efficacy (Supplementary Fig. 2).

**Fig. 2:**
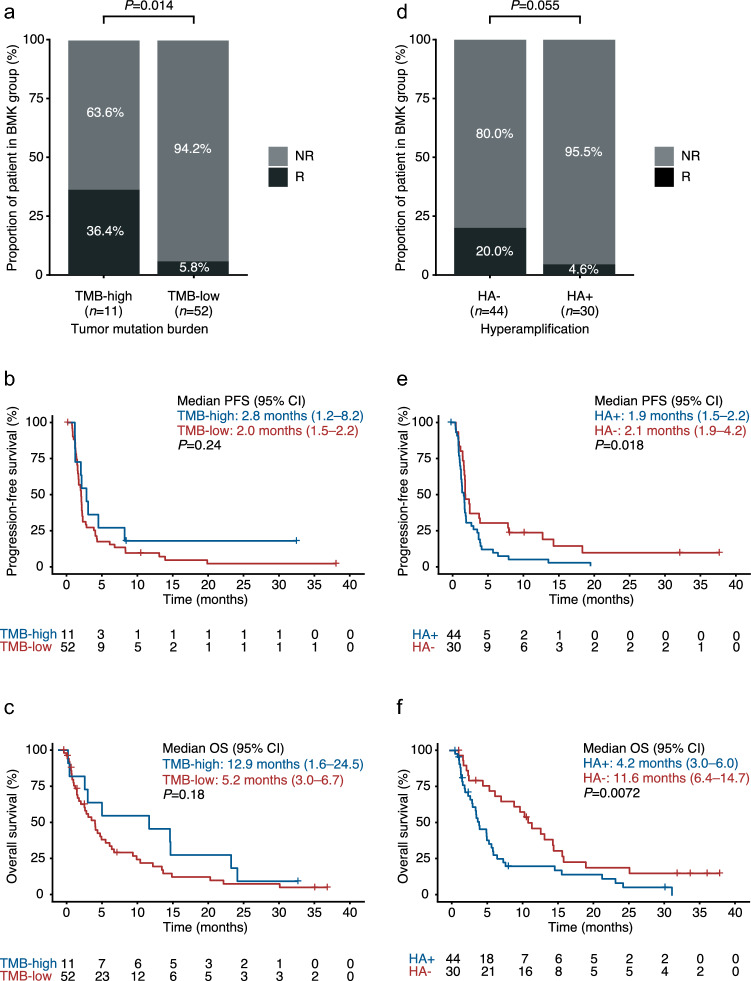
Association of somatic alteration with clinical outcome of tislelizumab monotherapy in the tislelizumab-treated GEA cohort. **a** Objective response rates according to TMB status, **b** Kaplan–Meier plot for PFS according to TMB status, **c** Kaplan–Meier plot for OS according to TMB status, **d** Objective response rates according to HA status, **e** Kaplan–Meier plot for PFS according to HA status, **f** Kaplan–Meier plot for OS according to HA status. *BMK* biomarker, *CI* confidence interval, *GEA* gastroesophageal adenocarcinoma, *HA* hyperamplification, *mOS* median OS, *mPFS* median PFS, *NR* non-responder, *OS* overall survival, *PFS* progression-free survival, *R* responder, *TMB* tumor mutational burden

To assess the role of somatic alteration in response to tislelizumab, the correlation between TMB status and clinical efficacy was analyzed. TMB-high tumors (17.5% of the BEP) demonstrated higher ORR compared with TMB-low tumors (ORR: 36.4 versus 5.8% [*P* = 0.014], respectively) and a trend toward longer mPFS (2.8 versus 2.0 months [*P* = 0.24], respectively) and mOS (12.9 versus 5.2 months [*P* = 0.18], respectively) (Fig. 2a–c).

Next, the possible impact of gene HA on the responsiveness to a PD-1 blockade was explored. HA + status was identified in 59.5% of the BEP and was strongly associated with poor clinical outcome, characterized by a lower ORR compared with HA − (4.6% versus 20.0% [*P* = 0.055], respectively), and shorter mPFS (1.9 versus 2.1 months [*P* = 0.018], respectively) and mOS (4.2 versus 11.6 months [*P* = 0.0072], respectively) (Fig. 2d–f).

**Fig. 3:**
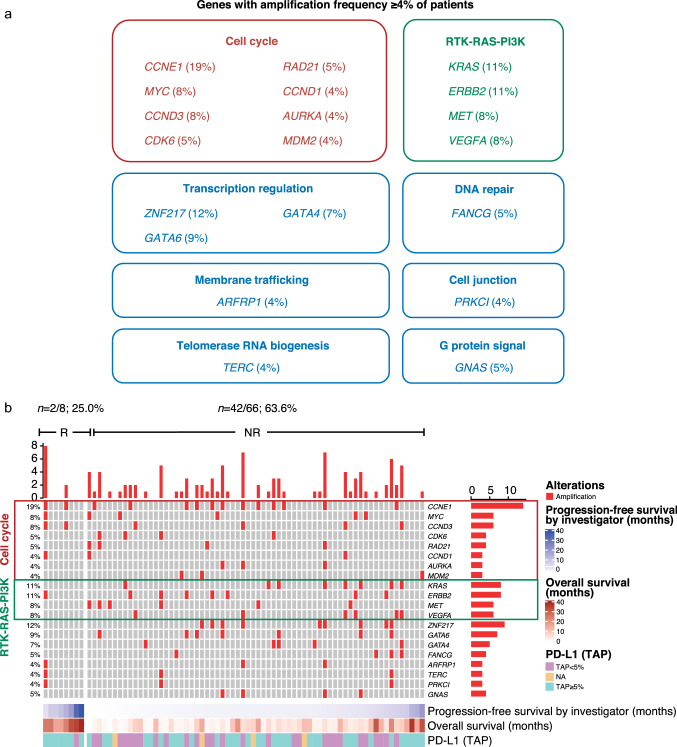
Characterization of genes with high amplification frequency in the tislelizumab-treated GEA cohort. **a** Genes with amplification frequency ≥ 4% of patients, **b** Gene HA landscape per patient categorized by response to tislelizumab monotherapy and ranked by PFS. *GEA* gastroesophageal adenocarcinoma, *HA* hyperamplification, *NA* not applicable, *NR* non-responder, *OS* overall survival, *PD-L1* programmed death-ligand 1, *PFS* progression-free survival, *R* responder

As shown in Fig. 3a, the most frequently hyperamplified genes were *CCNE1, ZNF217, KRAS, ERBB2, GATA6, MYC, CCND3, MET* and *VEGFA*. Among the 20 genes hyperamplified in ≥ 4% of patients, 40.0% (8/20) and 20.0% (4/20) were enriched in the cell cycle and RTK-RAS-PI3K pathways, respectively (Fig. 3a, b). When clinical outcomes were compared between patients with or without tumors that harbored hyperamplified genes specifically in the cell cycle or RTK-RAS-PI3K pathways, results were similar to the overall HA + versus HA − analyses, although trends tended to be non-significant (Supplementary Fig. 3a–f).

To further validate the role of HA during immune checkpoint blockade (ICB) treatment, biomarker data were analyzed from two independent cohorts: (i) patients with metastatic GC who were treated with pembrolizumab monotherapy; and (ii) patients with GI cancers treated with anti-PD(L)-1 and/or anti-CTLA-4 antibodies. Consistent with the finding for tislelizumab, patients with HA + status in these two independent validation cohorts also showed a lower response rate to ICB than those with HA − status (Supplementary Table 3). Furthermore, tumors with hyperamplified genes enriched in the cell cycle or RTK-RAS-PI3K pathways exhibited worse response to ICB than those without such hyperamplification in these independent cohorts (Supplementary Fig. 4a, b, Supplementary Tables 4, 5). Notably, hyperamplification of five fibroblast growth factors (FGFs), *FGF10*, *FGF19, FGF3, FGF4,* and *FGF23*, was found only in non-responders from these two cohorts (Supplementary Fig. 4a, b), suggesting that FGF/FGFR signaling may also contribute to poor responses to ICB. Meanwhile, HA + status was not associated with poor prognosis in patients treated with chemotherapy in the analysis of the TCGA-STAD cohort (Supplementary Fig. 5).

Taken together, these findings suggest that HA may be associated with poor response to ICB treatment in GEA and other GI cancers, and HA of cell cycle or RTK-RAS-PI3K related genes in particular may be an indicator for poor clinical response.

### HA combined with other biomarkers

Given the potential role of HA in responses to ICB, the possibility of integrating HA together with other biomarkers that may aid identification of patients most likely to respond to PD-1 inhibitors was explored. In our tislelizumab-treated population, no statistically significant interaction was found between HA status and PD-L1 expression status, IFN*γ* signature score, TIS score or TMB status for any of the clinical outcomes assessed (ORR, PFS or OS), suggesting an independent effect between HA status and other biomarkers (Supplementary Table 6). Furthermore, there was no association between HA status and individual interferon response-related gene expression (Supplementary Fig. 6). Across all assessed integrated biomarker groups, HA status and PD-L1 expression was the optimal combination for both prevalence and efficacy enrichment (Supplementary Table 7). Further analyses therefore focused on comparing clinical outcomes in patients stratified by joint utility of PD-L1 expression and HA status (*n* = 70).

**Fig. 4:**
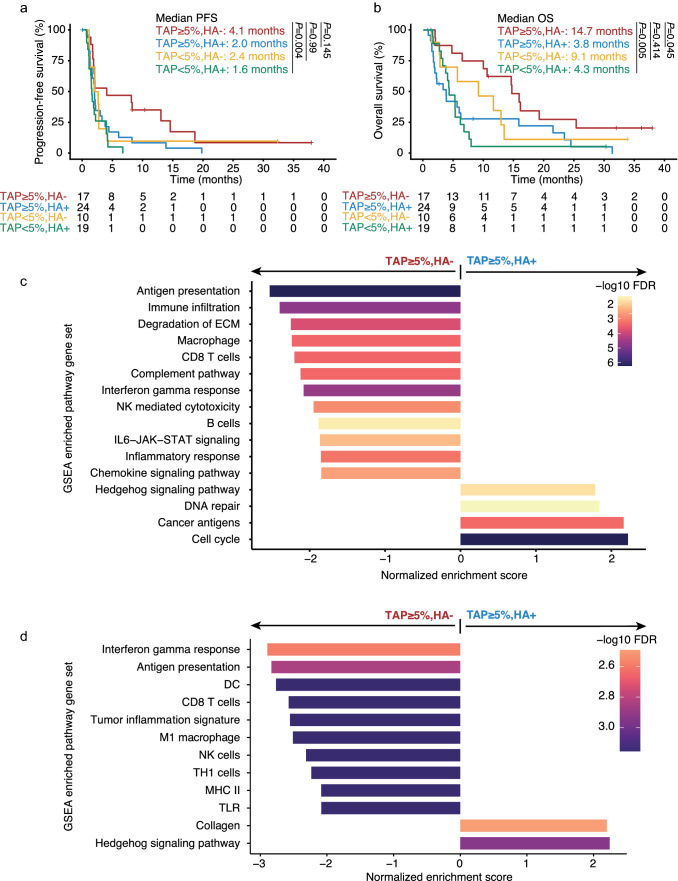
Association of joint PD-L1 status and HA with clinical outcome of tislelizumab monotherapy, and immune and tumor gene expression contexture in the tislelizumab-treated GEA cohort and pembrolizumab-treated independent GC validation cohort. **a** Kaplan–Meier plot for PFS by joint biomarker subgroup in the tislelizumab-treated GEA cohort, **b** Kaplan–Meier plot for OS by joint biomarker subgroup in the tislelizumab-treated GEA cohort, **c** Top rank gene expression signatures identified from TAP ≥ 5%, HA + versus TAP ≥ 5%, HA − subgroups in the tislelizumab-treated GEA cohort, **d** Top rank gene expression signatures identified from CPS ≥ 1, HA + versus CPS ≥ 1, HA − in the pembrolizumab-treated GC independent validation cohort. *CPS* combined positive score, *DC* dendritic cell, *DCR* disease control rate, *ECM* extracellular matrix, *FDR* false discovery rate, *GC* gastric cancer*, GEA* gastroesophageal adenocarcinoma, *GSEA* gene set enrichment analysis, *HA* hyperamplification, *IL* interleukin, *JAK* Janus kinase, *MHC* major histocompatibility complex, *NK* natural killer, *ORR* objective response rate, *OS* overall survival, *PD-L1* programmed death-ligand 1, *PFS* progression-free survival, *STAT,* signal transducer and activator of transcription, *TAP* Tumor Area Positivity, *TH* T helper, *TLR* toll-like receptor

Among the four subgroups, patients with TAP ≥ 5%, HA − tumors had the highest ORR and DCR (29.4 and 52.9%, respectively) (Table 1). Patients in the TAP ≥ 5%, HA − subgroup achieved an improved mPFS compared with those of the other three subgroups (4.1 months versus 2.0, 2.4 and 1.6 months for TAP ≥ 5%, HA + , TAP < 5%, HA − and TAP < 5%, HA + subgroups, respectively) (Fig. 4a). Similarly, mOS was also improved for the TAP ≥ 5%, HA − subgroup compared with the other subgroups (14.7 months versus 3.8, 9.1 and 4.3 months for subgroups TAP ≥ 5%, HA + , TAP < 5%, HA − and TAP < 5%, HA + subgroups respectively) (Fig. 4b). In the independent validation cohort of pembrolizumab-treated patients with GC, the subgroup with PD-L1 positive staining (CPS ≥ 1) and HA − had showed the most favorable clinical response to pembrolizumab compared with other subgroups, with an ORR of 62.5% and a DCR of 93.8% (Table 2). Whereas longer OS was not observed in patients with CD274^high^ plus HA − compared with other subgroups in the chemotherapy treated TCGA-STAD cohort (Supplementary Fig. 7).

**Table 1:** ORR and DCR by joint biomarker subgroup in the tislelizumab-treated GEA cohort

	Biomarker subgroup			
	TAP ≥ 5%, HA − (*n* = 17)	TAP ≥ 5%, HA + (*n* = 24)	TAP < 5%, HA − (*n* = 10)	TAP < 5%, HA + (*n* = 19)
Proportion of BEP group (*N* = 70), %	24.3	34.3	14.3	27.1
ORR, % (95% CI)	29.4 (10.3–56.0)	8.3 (1.0–27.0) *P* = 0.316	10.0 (0.3–44.5) *P* > 0.99	0 (0.0–17.6) *P* = 0.049
DCR, % (95% CI)	52.9 (27.8–77.0)	33.3 (15.6–55.3) *P* > 0.99	20.0 (2.5–55.6) *P* = 0.372	15.8 (3.4–39.6) *P* = 0.098

*P* values were from Fisher’s exact test. The Bonferroni method was used to adjust for multiplicity

*BEP* biomarker evaluable population, *CI* confidence interval, *DCR* disease control rate, *HA* hyperamplification, *GEA* gastroesophageal adenocarcinoma, *ORR* objective response rate, *TAP* tumor area positivity

**Table 2:** ORR and DCR by joint biomarker subgroup in the pembrolizumab-treated GC independent validation cohort

	Biomarker subgroup			
	CPS ≥ 1, HA − (*n* = 16)	CPS ≥ 1, HA + (*n* = 11)	CPS < 1, HA − (*n* = 9)	CPS < 1, HA + (*n* = 15)
Proportion of BEP group (*N* = 51), %	31.4	21.6	17.6	29.4
ORR, % (95% CI)	62.5 (35.4–84.8)	27.3 (6.0–61.0) *P* = 0.360	0 (0–33.6) *P* = 0.008	0 (0–21.8) *P* = 0.001
DCR, % (95% CI)	93.8 (69.8–99.8)	72.7 (39.0–94.0) *P* = 0.819	33.3 (7.5–70.1) *P* = 0.009	33.3 (11.8–61.6) *P* = 0.002

*P* values were from Fisher’s exact test. The Bonferroni method was used to adjust for multiplicity

*BEP* biomarker evaluable population, *CI* confidence interval, *CPS* combined positive score, *DCR* disease control rate, *HA* hyperamplification, *GC* gastric cancer, *ORR* objective response rate

### Comparison of gene expression profiles in PD-L1 TAP ≥ 5% tumors with or without HA

The final analyses explored how HA affects the characteristics of immune and tumor gene expression in the PD-L1 positive tumors. Gene set enrichment analysis showed elevated expression of pathways relating to immune cells (including CD8 *T* cells, NK cells, and macrophages), IFN*γ*, and an antigen presenting gene signature in PD-L1 positive, HA − tumors in both the tislelizumab-treated GEA cohort and pembrolizumab-treated GC cohort (Fig. 4c, d). In addition, the IHC CD8 + density analysis in the subset of tislelizumab-treated patients from NCT02407990 revealed a higher tumor and stromal CD8 + *T* cell abundance in TAP ≥ 5%, HA − versus TAP ≥ 5%, HA + subgroups (Supplementary Fig. 8).

Differential gene expression analysis in the overall tislelizumab-treated GEA cohort identified genes highly expressed in TAP ≥ 5%, HA + tumors, including genes with functions in cell cycle regulatory (such as aurora kinase A [*AURKA*], ubiquitin-conjugating enzyme 2C [*UBE2C*], *CDC20* and *MYBL2*), DNA repair (such as *RAD54L* and *FANCD2*), and hedgehog signaling (such as *WNT5A* and IHH) pathways, or with functions as cancer antigens (Supplementary Fig. 9).

## Discussion

Tislelizumab demonstrated anti-tumor activity in patients with GEA during this long-term follow-up of two early phase clinical trials. The present study explored the association of biomarkers including PD-L1 status, IFN*γ* gene signature expression, TIS score, TMB, and gene hyperamplification with tislelizumab clinical efficacy. Aligned with previous studies, our investigation showed an association between PD-L1 expression, IFN*γ* gene signature expression, TIS score and clinical efficacy of checkpoint blockade immunotherapy [9, 26, 36]. However, the clinical benefit enrichment with these established biomarkers is still limited, highlighting the need of other biomarkers to identify patients who will benefit from PD-1 inhibition in GEA.

In somatic alteration analysis, TMB-high tumors showed a higher response to tislelizumab compared with TMB-low tumors; however, the low prevalence of TMB-high tumors may restrict the application of TMB status as a biomarker of response in GEA. Beyond TMB, HA (defined by CN > 5) was found to be broadly distributed in GEA and GI cohorts, especially in non-responders, which suggested its potential role in PD-1 inhibitor resistance. Consistent with our findings in this study, results from the non-ICB-treated Memorial Sloan Kettering pan-cancer cohort revealed that a large portion of gastrointestinal stromal tumors are TMB-low and CNA-high [37], which suggests gene CN change is an important feature of gastric tumors and may play key roles in regulating ICB response.

Our study found HA mainly occurred in genes involved in the cell cycle and RTK-RAS-PI3K pathways, which are known to be involved in tumorigenesis. Previous studies have reported that amplifications of cell cycle driver genes and RTK-RAS-PI3K pathway genes, such as CCND1, MDM2, MET and VEGF-A, were associated with a lack of response to or poor survival benefit with anti-PD(L)-1 treatment [38–44]. In addition, reduced interferon response, low *T* cell abundance, poor *T* cell activity and a more immunosuppressive tumor microenvironment (enriched TGF-β signaling, hypoxia signaling) were observed in tumors with amplified genes in these two pathways, suggesting that hyperamplification in cell cycle/RTK-RAS-PI3K pathways may be a key point to drive anti-PD(L)-1 resistance through inducing both an immunosuppressive tumor microenvironment and malignancy hallmarks [40, 41, 44–47]. However, HA was not associated with poor outcomes in patients treated with chemotherapy in the independent TCGA-STAD cohort, suggesting that this association may be specific to checkpoint inhibition.

The potential predictive ability of an integrated biomarker combining PD-L1 positive status without HA (TAP ≥ 5%, HA −) was subsequently assessed and found to better correlate with improved tislelizumab clinical efficacy than TAP ≥ 5% or HA − alone. Samples from the TAP ≥ 5%, HA − subgroup exhibited an inflamed gene signature, including genes linked to immune cell infiltration (including* T*-, B-, and NK-cells, and macrophages), IFN*γ* signaling, and antigen presentation. This finding points to the importance of both adaptive and innate immunity in the response to tislelizumab. In contrast, samples from the TAP ≥ 5%, HA + subgroup exhibited a less inflamed gene signature together with highly expressed cell cycle, cancer antigen, and DNA repair signatures, and in this subgroup fewer tumors responded to tislelizumab treatment. Taken together, this observation indicates an interaction between HA and the tumor immune microenvironment, and provides a potential explanation why tumors in the TAP ≥ 5%, HA + subgroup did not respond to PD-1 therapy despite being PD-L1 positive. To our knowledge, this is the first report of the utility of a combined PD-L1 expression and HA biomarker for identifying patients who may be more likely to benefit from PD-1 inhibition.

Through differentially expressed gene analysis, some potential druggable genes that were highly expressed in TAP ≥ 5%, HA + compared with TAP ≥ 5%, HA − tumors were observed, such as *AURKA* and *UBE2C*. Previous studies have shown that overexpression of *AURKA* and *UBE2C* frequently occurred in GC, promoting cancer cell proliferation or epithelial-mesenchymal transition [48–50]. A selective inhibitor of AURKA (alisertib) has shown a manageable safety profile and an ORR of 9% among 47 patients with GEA [51].

This exploratory analysis has limitations. Firstly, the robustness of our analyses and the clinical utilization of the studied biomarkers is limited by small sample size and few responders. Secondly, the single-arm design of trials NCT02407990 and NCT04068519 prevented us from determining whether the identified biomarkers were predictive of treatment response or prognostic. Thirdly, the genomic profile platform in our study and the PD-L1 expression assay differed from those used in the independent cohorts and TCGA dataset. Fourth, while analyses based on Lauren histological subtypes may have provided further insights, Lauren classification data were not available from the two tislelizumab trials. Given the above limitations, exploratory biomarkers reported in this study will be further validated in larger, randomized studies [52].

In conclusion, our analysis suggests PD-L1 positive status, IFN*γ* gene signature expression, TIS score, and TMB-high status are positively associated with tislelizumab clinical benefit in GEA tumors, whereas HA was associated with worse clinical outcomes. Combining PD-L1 positivity and lack of HA as an integrated biomarker may better identify patients who are more likely to respond to PD-1 blockade.

## Supplementary Information

Below is the link to the electronic supplementary material.

Supplementary file1 (DOCX 1234 KB)
