# Supplementary material for: The combination of gene hyperamplification and PD-L1 expression as a biomarker for the clinical benefit of tislelizumab in gastric/gastroesophageal junction adenocarcinoma
Source: Gastric Cancer. 2022 Jul 2;25(5):943–55. doi: 10.1007/s10120-022-01308-7 (PMC9365737; doi:10.1007/s10120-022-01308-7)
Supplement: Supplementary file 1 — Supplementary file1 (DOCX 1234 KB) [file 10120_2022_1308_MOESM1_ESM.docx]

Supplementary Appendix

**Supplementary Table 1** Baseline characteristics and clinical outcome of tislelizumab monotherapy in in the overall pooled tislelizumab-treated GEA cohort, and in the GEA cohorts from the individual studies.

|  |  | **Overall  GEA cohort (*N*=105)** | **NCT02407990 GEA cohort (*n*=81)** | **NCT04068519  GEA cohort (*n*=24)** |
| --- | --- | --- | --- | --- |
| Age, n (%) | <65 years | 66 (62.9) | 48 (59.3) | 18 (75.0) |
|  | ≥65 years | 39 (37.1) | 33 (40.7) | 6 (25.0) |
| Sex, n (%) | Male | 72 (68.6) | 54 (66.7) | 18 (75.0) |
|  | Female | 33 (31.4) | 27 (33.3) | 6 (25.0) |
| Race, n (%) | Asian | 50 (47.6) | 26 (32.1) | 24 (100.0) |
|  | White | 45 (42.9) | 45 (55.6) | 0 (0.0) |
|  | Other | 10 (9.5) | 10 (12.3) | 0 (0.0) |
| Histology at baseline, n (%) | Poorly differentiated | 49 (76.6) | 35 (71.4) | 14 (93.3) |
|  | Well differentiated | 2 (3.1) | 2 (4.1) | 0 (0.0) |
|  | Unknown | 13 (20.3) | 12 (24.5) | 1 (6.7) |
| Tumor stage at baseline, n (%) | Stage III | 5 (4.8) | 5 ( 6.2) | 0 (0.0) |
|  | Stage IV | 100 (95.2) | 76 (93.8) | 24 (100.0) |
| Lines of prior systemic anticancer therapy, n (%) | 1 | 13 (12.4) | 13 (16.0) | 0 (0.0) |
|  | 2 | 39 (37.1) | 29 (35.8) | 10 (41.7) |
|  | ≥3 | 53 (50.5) | 39 (48.1) | 14 (58.3) |
| Histology type, n (%) | Adenocarcinoma | 102 (97.1) | 78 (96.3) | 24 (100.0) |
|  | Mixed adeno/squamous carcinoma | 1 (1.0) | 1 (1.2) | 0 (0.0) |
|  | Others | 2 (1.9) | 2 (2.5) | 0 (0.0) |
| Tumor site, n (%) | GC/GEJ | 78 (74.3) | 54 (66.7) | 24 (100.0) |
|  | EAC | 27 (25.7) | 27 (33.3) | 0 (0.0) |
| Clinical response | | | | |
| ORR, % (95% CI) | | 11.4  (6.1–19.1) | 9.9  (4.4–18.5) | 16.67  (4.74–37.38) |
| DCR, % (95% CI) | | 29.5  (21.0–39.2) | 30.9  (21.1–42.1) | 25.0  (9.77–46.71) |
| mPFS, months (95% CI) | | 2.0  (1.9–2.1) | 2.0  (1.8–2.1) | 2.1  (1.94–4.01) |
| mOS, months (95% CI) | | 5.7  (4.3–8.6) | 6.0  (4.2–9.1) | 4.7  (2.4–14.6) |
| Median follow-up, months (95% CI) | | 32.8  (32.0–38.0) | 32.0  (30.3–38.0) | 32.8  (NR–NR) |

*CI* confidence interval, *DCR* disease control rate, *EAC* esophageal adenocarcinoma, *GC/GEJ* gastric/gastroesophageal junction, *GEA* gastroesophageal adenocarcinoma, *mOS* median overall survival, *mPFS* median progression-free survival, *NR* non-responder, *ORR* objective response rate

**Supplementary Table 2** Baseline characteristics and clinical outcome of tislelizumab monotherapy in biomarker evaluable populations and the overall population in the tislelizumab-treated GEA cohort.

|  | |  | **Biomarker evaluable populations** | | | |
| --- | --- | --- | --- | --- | --- | --- |
| **Characteristic** | | **Overall**  **(*N*=105)** | **PD-L1 evaluable**  **(*n*=92)** | **GEP evaluable**  **(*n*=80)** | **Gene alteration evaluable**  **(*n*=74)** | **TMB**  **evaluable**  **(*n*=63)** |
| Age, n (%) | <65 years | 66 (62.9) | 60 (65.2) | 52 (65.0) | 50 (67.6) | 43 (68.3) |
|  | ≥65 years | 39 (37.1) | 32 (34.8) | 28 (35.0) | 24 (32.4) | 20 (31.7) |
| Sex, n (%) | Female | 33 (31.4) | 30 (32.6) | 24 (30.0) | 29 (39.2) | 25 (39.7) |
|  | Male | 72 (68.6) | 62 (67.4) | 56 (70.0) | 45 (60.8) | 38 (60.3) |
| Race, n (%) | Asian | 50 (47.6) | 46 (50.0) | 37 (46.2) | 35 (47.3) | 31 (49.2) |
|  | White | 45 (42.9) | 39 (42.4) | 36 (45.0) | 30 (40.5) | 25 (39.7) |
|  | Other | 10 (9.5) | 7 (7.6) | 7 (8.8) | 9 (12.2) | 7 (11.1) |
| Histology at baseline, n (%) | Poorly differentiated | 49 (76.6) | 43 (76.8) | 35 (77.8) | 34 (75.6) | 30 (76.9) |
|  | Well differentiated | 2 (3.1) | 2 (3.6) | 2 (4.4) | 2 (4.4) | 2 (5.1) |
|  | Unknown | 13 (20.3) | 11 (19.6) | 8 (17.8) | 9 (20.0) | 7 (17.9) |
| Tumor site, n (%) | EAC | 27 (25.7) | 26 (28.3) | 24 (30.0) | 17 (23.0) | 12 (19.0) |
|  | GC/GEJ | 78 (74.3) | 66 (71.7) | 56 (70.0) | 57 (77.0) | 51 (81.0) |
| Histology type, n (%) | Adenocarcinoma | 102 (97.1) | 89 (96.7) | 78 (97.5) | 72 (97.3) | 61 (96.8) |
|  | Mixed adeno/squamous carcinoma | 1 (1.0) | 1 (1.1) | 1 (1.2) | 1 (1.4) | 1 (1.6) |
|  | Others | 2 (1.9) | 2 (2.2) | 1 (1.2) | 1 (1.4) | 1 (1.6) |
| Tumor stage at baseline, n (%) | Stage III | 5 (4.8) | 4 (4.3) | 4 (5.0) | 3 (4.1) | 3 (4.8) |
|  | Stage IV | 100 (95.2) | 88 (95.7) | 76 (95.0) | 71 (95.9) | 60 (95.2) |
| Lines of prior systemic anticancer therapy, n (%) | 1 | 13 (12.4) | 9 (9.8) | 9 (11.2) | 7 (9.5) | 7 (11.1) |
|  | 2 | 39 (37.1) | 33 (35.9) | 31 (38.8) | 26 (35.1) | 22 (34.9) |
|  | ≥3 | 53 (50.5) | 50 (54.3) | 40 (50.0) | 41 (55.4) | 34 (54.0) |
| Clinical response | | | | | | |
| ORR, % (95% CI) | | 11.4  (6.1–19.1) | 12.0  (6.1–20.4) | 11.3  (5.3–20.3) | 10.8  (4.8–20.2) | 11.1  (4.6–21.6) |
| DCR, % (95% CI) | | 29.5  (21.0–39.2) | 29.4  (20.3–39.8) | 27.5  (18.1–38.6) | 29.7  (19.7–41.5) | 27.0  (16.6–39.7) |
| mPFS, months (95% CI) | | 2.0  (1.9–2.1) | 2.0  (1.9–2.2) | 2.0  (1.7–2.1) | 2.0  (1.7–2.2) | 2.0  (1.6–2.2) |
| mOS, months (95% CI)) | | 5.7  (4.3–8.6) | 5.5  (4.0–6.7) | 5.3  (3.9–7.6) | 6.0  (4.2–9.1) | 5.3  (3.8–7.3) |
| Median follow-up, months (95% CI) | | 32.8  (32.0–38.0) | 32.8  (30.3–36.2) | 33.8  (32.0–36.2) | 33.8  (30.3–36.2) | 36.2  (33.8–NR) |

*DCR* disease control rate, *EAC* esophageal adenocarcinoma, *GC/GEJ* gastric/gastroesophageal junction, *GEP* gene expression profile, *mOS* median overall survival, *mPFS* median progression-free survival, *NR* non-responder, *ORR* objective response rate, *PD-L1* programmed death-ligand 1, *TMB* tumor mutational burden

**Supplementary Table 3** Clinical response in patients with HA+ versus HA- in the immune checkpoint inhibitor-treated GI cancer independent validation cohort.

|  | **Biomarker subgroup** | |
| --- | --- | --- |
|  | **HA-**  **(*n*=54)** | **HA+**  **(*n*=28)** |
| CR, n | 1 | 0 |
| PR, n | 13 | 3 |
| SD, n | 13 | 7 |
| PD, n | 27 | 18 |
| ORR, % (95% CI) | 25.9 (15.0–39.7) | 10.7 (2.3–28.2) |
| DCB, % (95% CI) | 42.6 (29.2–56.8) | 25.0 (10.7–44.9) |

Patients in this cohort received anti-PD(L)-1 and/or anti-CTLA-4 antibodies. DCB was defined as CR, PR, or SD lasting for ≥24 weeks.
*CI* confidence interval, *CR* complete response, *DCB* durable clinical benefit, *HA* hyperamplification, *GI* gastrointestinal, *ORR* objective response rate, *PD* progressive disease, *PR* partial response, *SD* stable disease

**Supplementary Table 4** Clinical response in patients with hyperamplified genes in cell cycle or RTK-RAS-PI3K pathways in the pembrolizumab-treated GC independent validation cohort.

|  | **Biomarker subgroup** | | | |
| --- | --- | --- | --- | --- |
|  | **Cell cycle pathway** | | **RTK-RAS-PI3K pathway** | |
|  | **HA-**  **(*n*=40)** | **HA+**  **(*n*=15)** | **HA-**  **(*n*=42)** | **HA+**  **(*n*=13)** |
| ORR, % (95% CI) | 30.0  (16.6–46.5) | 13.3  (1.7–40.5) | 31.0  (17.6–47.1) | 7.7  (0.2–36.0) |
| DCR, % (95% CI) | 65.0  (48.3–79.4) | 46.7  (21.3–73.4) | 61.9  (45.6–76.4) | 53.8  (25.1–80.8) |

DCR was defined as the proportion of patients who achieved complete response, partial response, or stable disease.
*CI* confidence interval, *DCR* disease control rate, *GC* gastric cancer, *HA* hyperamplification, *ORR* objective response rate

**Supplementary Table 5** Clinical response in patients with hyperamplified genes in cell cycle or RTK-RAS-PI3K pathways in the immune checkpoint inhibitor-treated GI cancer independent validation cohort.

|  | **Biomarker subgroup** | | | |
| --- | --- | --- | --- | --- |
|  | **Cell cycle pathway** | | **RTK-RAS-PI3K pathway** | |
|  | **HA-**  **(*n*=67)** | **HA+**  **(*n*=15)** | **HA-**  **(*n*=69)** | **HA+**  **(*n*=13)** |
| DCB, % (95% CI) | 40.3 (28.5–53.0) | 20.0 (4.3–48.1) | 42.0 (30.2–54.5) | 7.7 (0.2–36.0) |

Patients in this cohort received anti-PD(L)-1 and/or anti-CTLA-4 antibodies. DCB was defined as CR, PR, or SD lasting for ≥24 weeks.
*CI* confidence interval, *CR* complete response*, DCB* durable clinical benefit, *HA* hyperamplification, *GI* gastrointestinal*, PR* partial response, *SD* stable disease

**Supplementary Table 6** Analysis of interaction between HA and PD-L1, IFNꝩ, TIS or TMB in the tislelizumab-treated GEA cohort.

| Biomarker group | Outcome | HA | Biomarker subgroup | OR or HR* | 95% CI | *P*-value | Interaction  *P-*value |
| --- | --- | --- | --- | --- | --- | --- | --- |
| HA vs PD-L1 | ORR | HA+ vs HA- | Overall | OR: 0.00 | 0–infinite | 0.994 | 0.995 |
|  |  | HA+ vs HA- | TAP<5% | OR: 0.00 | 0–infinite | 0.994 |  |
|  |  | HA+ vs HA- | TAP≥5% | OR: 0.22 | 0.04–1.30 | 0.094 |  |
|  | PFS | HA+ vs HA- | Overall | HR: 1.92 | 1.13–3.26 | 0.017 | 0.897 |
|  |  | HA+ vs HA- | TAP<5% | HR: 1.85 | 0.82–4.16 | 0.136 |  |
|  |  | HA+ vs HA- | TAP≥5% | HR: 1.98 | 1.01–3.90 | 0.047 |  |
|  | OS | HA+ vs HA- | Overall | HR: 2.08 | 1.18–3.67 | 0.011 | 0.471 |
|  |  | HA+ vs HA- | TAP<5% | HR: 1.70 | 0.72–4.03 | 0.228 |  |
|  |  | HA+ vs HA- | TAP≥5% | HR: 2.56 | 1.25–5.23 | 0.010 |  |
| HA vs IFNꝩ signature | ORR | HA+ vs HA- | Overall | OR: 0.00 | 0–infinite | 0.994 | 0.994 |
|  |  | HA+ vs HA- | IFNꝩ-low | OR: 0.00 | 0–infinite | 0.994 |  |
|  |  | HA+ vs HA- | IFNꝩ-high | OR: 0.42 | 0.06–2.95 | 0.380 |  |
|  | PFS | HA+ vs HA- | Overall | HR: 1.71 | 0.98–3.01 | 0.061 | 0.112 |
|  |  | HA+ vs HA- | IFNꝩ-low | HR: 2.71 | 1.16–6.29 | 0.021 |  |
|  |  | HA+ vs HA- | IFNꝩ-high | HR: 1.08 | 0.51–2.29 | 0.833 |  |
|  | OS | HA+ vs HA- | Overall | HR: 2.51 | 1.37–4.61 | 0.003 | 0.514 |
|  |  | HA+ vs HA- | IFNꝩ-low | HR: 3.06 | 1.25–7.51 | 0.015 |  |
|  |  | HA+ vs HA- | IFNꝩ-high | HR: 2.06 | 0.93–4.58 | 0.076 |  |
| HA vs TIS signature | ORR | HA+ vs HA- | Overall | OR: 0.29 | 0.04–1.91 | 0.197 | 0.881 |
|  |  | HA+ vs HA- | TIS-low | OR: 0.25 | 0.01–4.60 | 0.351 |  |
|  |  | HA+ vs HA- | TIS-high | OR: 0.33 | 0.03–3.68 | 0.370 |  |
|  | PFS | HA+ vs HA- | Overall | HR: 1.68 | 0.93–3.04 | 0.088 | 0.176 |
|  |  | HA+ vs HA- | TIS-low | HR: 2.56 | 1.02–6.38 | 0.045 |  |
|  |  | HA+ vs HA- | TIS-high | HR: 1.10 | 0.50–2.41 | 0.809 |  |
|  | OS | HA+ vs HA- | Overall | HR: 2.42 | 1.27–4.61 | 0.007 | 0.782 |
|  |  | HA+ vs HA- | TIS-low | HR: 2.64 | 0.99–7.05 | 0.052 |  |
|  |  | HA+ vs HA- | TIS-high | HR: 2.21 | 0.97–5.05 | 0.060 |  |
| HA vs TMB | ORR | HA+ vs HA- | Overall | OR: 0.12 | 0.02–0.84 | 0.033 | 0.466 |
|  |  | HA+ vs HA- | TMB-low | OR: 0.24 | 0.02–2.88 | 0.262 |  |
|  |  | HA+ vs HA- | TMB-high | OR: 0.06 | 0.00–1.23 | 0.068 |  |
|  | PFS | HA+ vs HA- | Overall | HR: 3.09 | 1.32–7.28 | 0.010 | 0.082 |
|  |  | HA+ vs HA- | TMB-low | HR: 1.46 | 0.80–2.66 | 0.218 |  |
|  |  | HA+ vs HA- | TMB-high | HR: 6.56 | 1.34–32.25 | 0.021 |  |
|  | OS | HA+ vs HA- | Overall | HR: 2.40 | 1.12–5.14 | 0.024 | 0.362 |
|  |  | HA+ vs HA- | TMB-low | HR: 1.70 | 0.89–3.24 | 0.110 |  |
|  |  | HA+ vs HA- | TMB-high | HR: 3.41 | 0.87–13.33 | 0.078 |  |

*OR for response; HR for PFS and OS. *CI* confidence interval, *GEA* gastroesophageal adenocarcinoma, *HA* hyperamplification, *HR* hazard ratio, *IFNꝩ* interferon gamma, *OR* odds ratio, *OS* overall survival, *PD-L1* programmed death-ligand 1, *PFS* progression-free survival, *TAP* Tumor Area Positivity, *TIS* T-cell-inflamed signature, *TMB* tumor mutational burden

**Supplementary Table 7** Prevalence and clinical outcomes of tislelizumab monotherapy in combined biomarker subgroups that enhanced clinical activity versus the remainder of the population, in the tislelizumab-treated GEA cohort.

| **Biomarker subgroup** | **Prevalence**  % (*n/N*) | **ORR**  % (95% CI) | **mPFS**  months (95% CI) | **mOS**  months (95% CI) |
| --- | --- | --- | --- | --- |
| TAP≥5%,HA- | 24.3 (17/70) | 29.4 (10.3–56.0) | 4.1 (1.8–13.0) | 14.7 (6.4–25.4) |
| IFNꝩ-high,HA- | 21.7 (13/60) | 23.1 (5.0–53.8) | 1.9 (1.2–8.1) | 11.1 (4.7–25.4) |
| TIS-high,HA- | 25.0 (15/60) | 20.0 (4.3–48.1) | 2.1 (1.2–2.8) | 11.1 (4.7–19.2) |
| TMB-high,HA- | 6.3 (4/63) | 75.0 (19.4–99.4) | 8.1 (2.8–NR) | 20.6 (12.9–NR) |

*CI* confidence interval, *GEA* gastroesophageal adenocarcinoma, *HA* hyperamplification, *IFNꝩ* interferon gamma, *NR* not reached, *ORR* objective response rate, *OS* overall survival, *PFS* progression-free survival, *TAP* Tumor Area Positivity, *TIS* T-cell-inflamed signature, *TMB* tumor mutational burden

**Supplementary Fig. 1** TMB concordance between F1CDx and OncoScreen 520 in advanced solid tumors (N=54) from study NCT02407990.


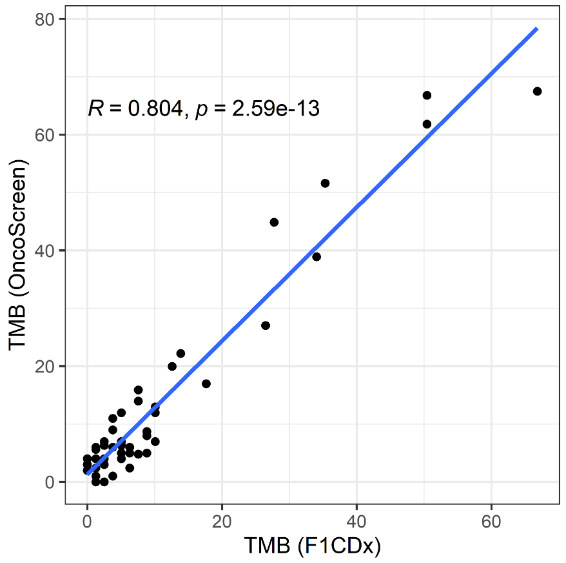


TMB, tumor mutational burden

**Supplementary Fig. 2** The association of TIS score with clinical outcome of tislelizumab monotherapy in the tislelizumab-treated GEA cohort. **a** Objective response rates according to TIS score status; **b** Kapan–Meier plot for PFS according to TIS score status; **c** Kapan–Meier plot for OS according to TIS score status.


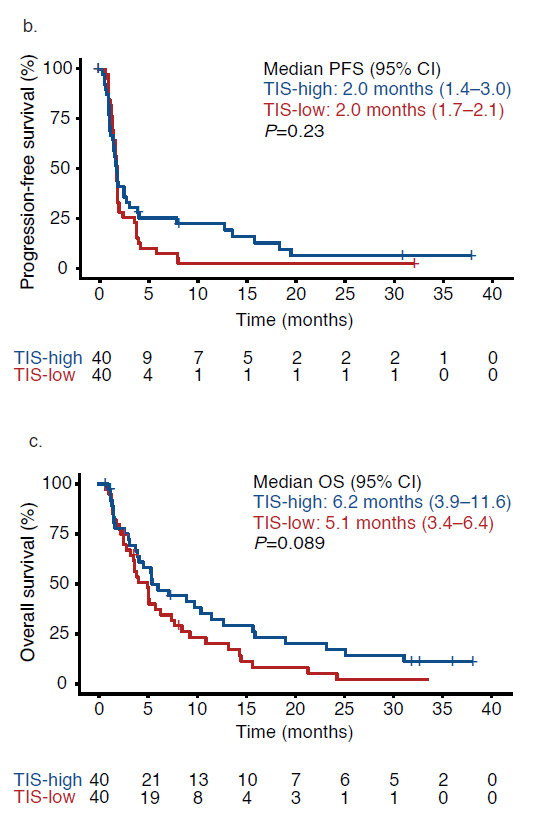

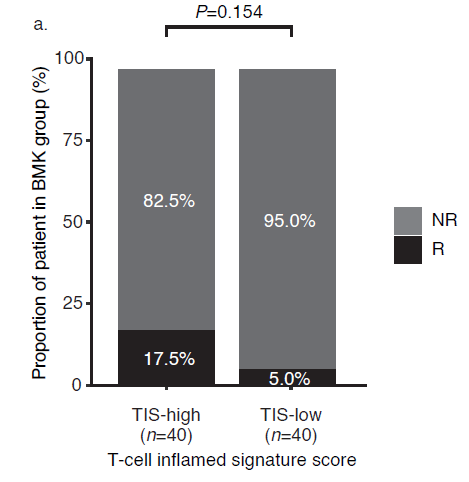


*BMK* biomarker, *CI* confidence interval, *GEA* gastroesophageal adenocarcinoma, *mOS* median OS, *mPFS* median PFS, *NR* non-responder, *OS* overall survival, *PFS* progression-free survival, *R* responder, *TIS* T-cell inflamed signature

**Supplementary Fig. 3** The association of gene HA in the cell cycle and RTK-RAS-PI3K pathways with clinical outcome of tislelizumab monotherapy in the tislelizumab-treated GEA cohort. **a** Objective response rates according to cell cycle HA status; **b** Kapan–Meier plot for PFS according to according to cell cycle HA status; **c** Kapan–Meier plot for OS according to cell cycle HA status; **d** Objective response rates according to RTK-RAS-PI3K HA status; **e** Kapan–Meier plot for PFS according to according to RTK-RAS-PI3K HA status; **f** Kapan–Meier plot for OS according to RTK-RAS-PI3K HA status.


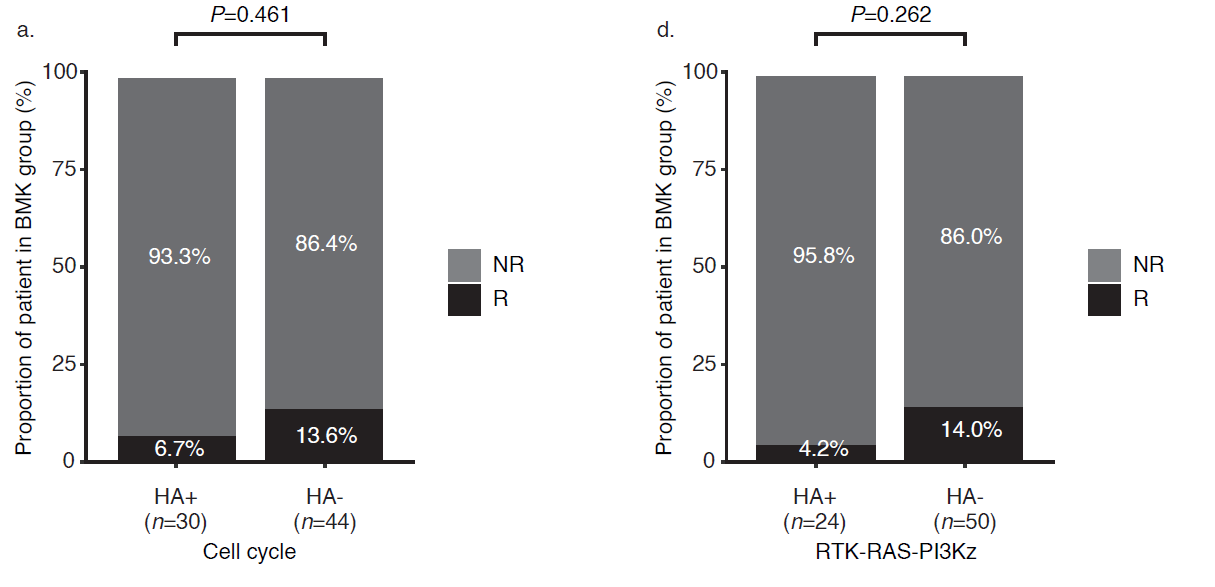


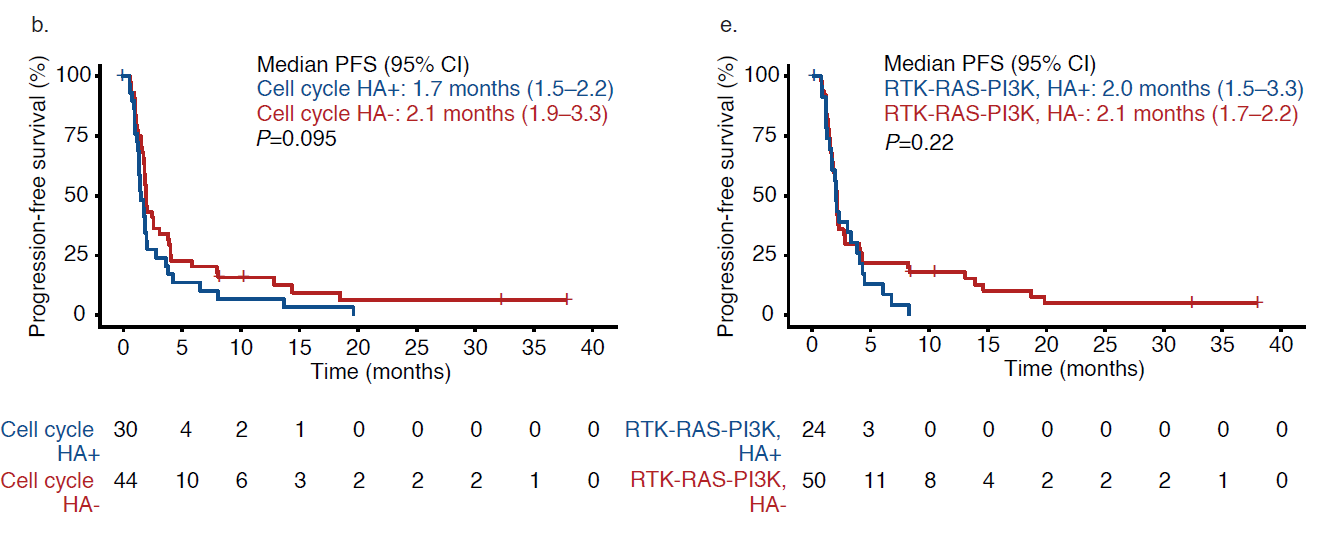


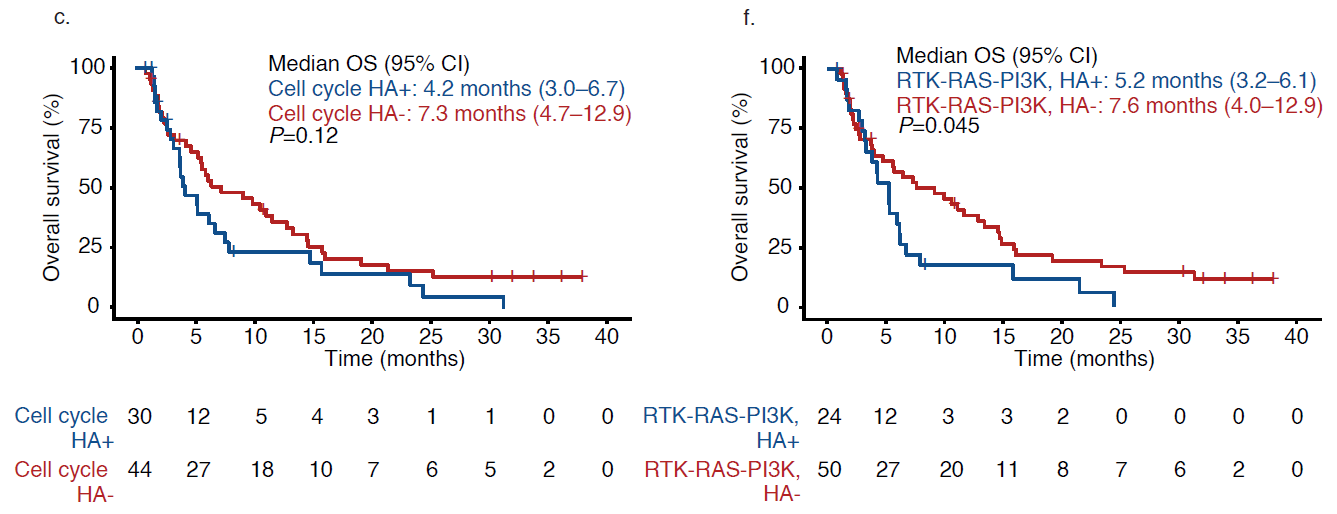


*BMK* biomarker, *CI* confidence interval, *GEA* gastroesophageal adenocarcinoma, *mOS* median OS, *mPFS* median PFS, *NR* non-responder, *OS* overall survival, *PFS* progression-free survival, *R* responder


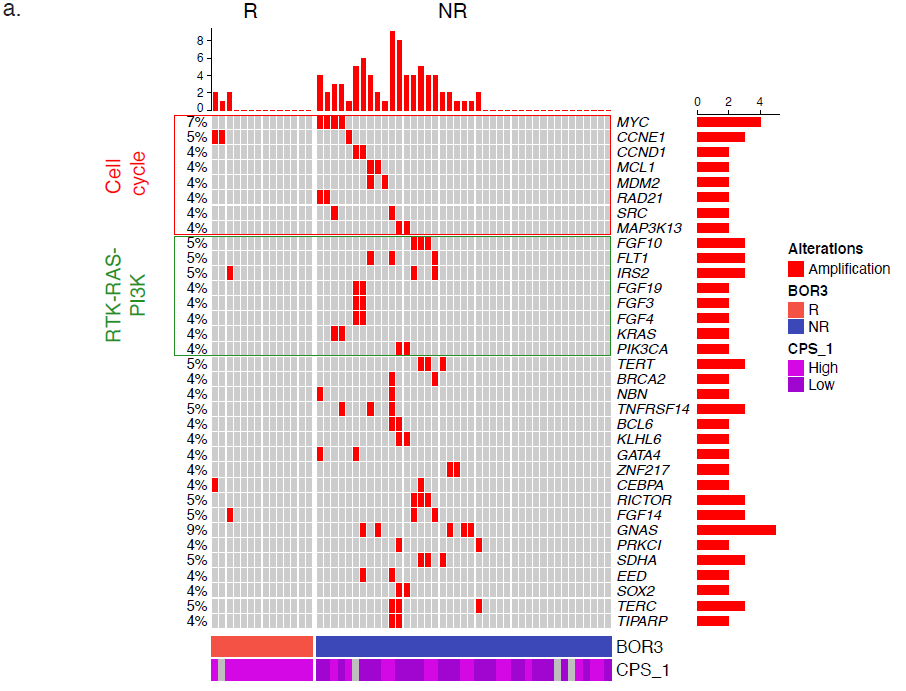
**Supplementary Fig. 4** Hyperamplified genes enriched in cell cycle or RTK-RAS-PI3K pathways in two independent validation cohorts. **a** Genes HA landscape categorized by response in the pembrolizumab-treated GC independent validation cohort; **b** Genes HA landscape categorized by response in the immune checkpoint inhibitor-treated GI cancer independent validation cohort.


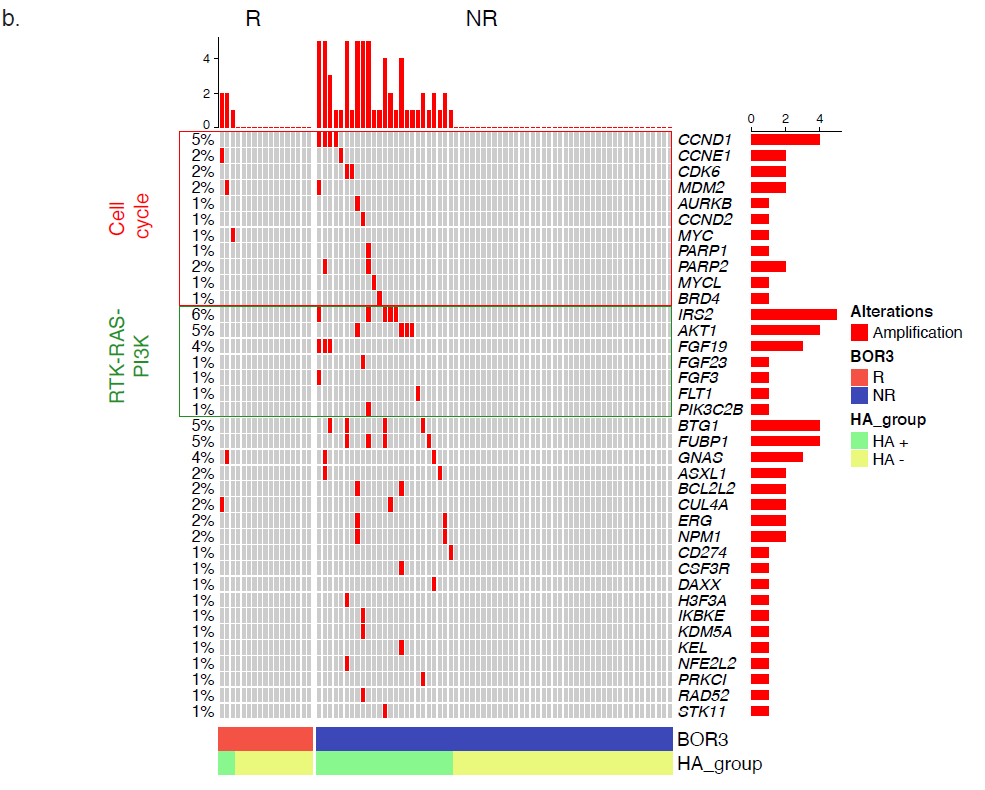


Patients in the immune checkpoint inhibitor-treated GI cancer independent validation cohort received anti-PD(L)-1 and/or anti-CTLA-4 antibodies.  *CPS* combined positive score, *HA* hyperamplification, *GC* gastric cancer, *GI* gastrointestinal*,* *NE* not evaluable, *NR* non-responder, *PD-L1* programmed death-ligand 1, *R* responder

**Supplementary Fig. 5** The association of gene HA with clinical outcome in the chemotherapy-treated TCGA-STAD independent validation cohort.


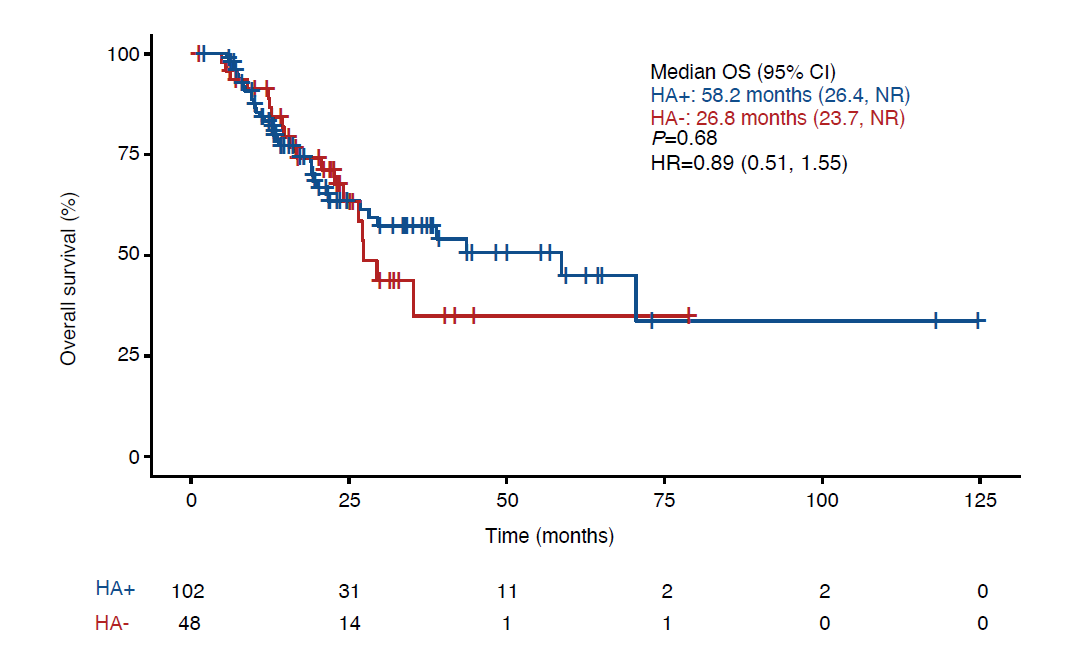


Figure presents a Kaplan-Meier plot for OS according to HA status.
*CI* confidence interval, *HA* hyperamplification, *HR* hazard ratio, *NR* not reached, *OS* overall survival, *STAD* stomach adenocarcinoma, *TCGA* The Cancer Genome Atlas

**Supplementary Fig. 6** Analysis of interaction between HA status and individual interferon response-related gene expression in the tislelizumab-treated GEA cohort. **a** HA and interferon-related gene expression; **b** HA and PD-L1 expression.


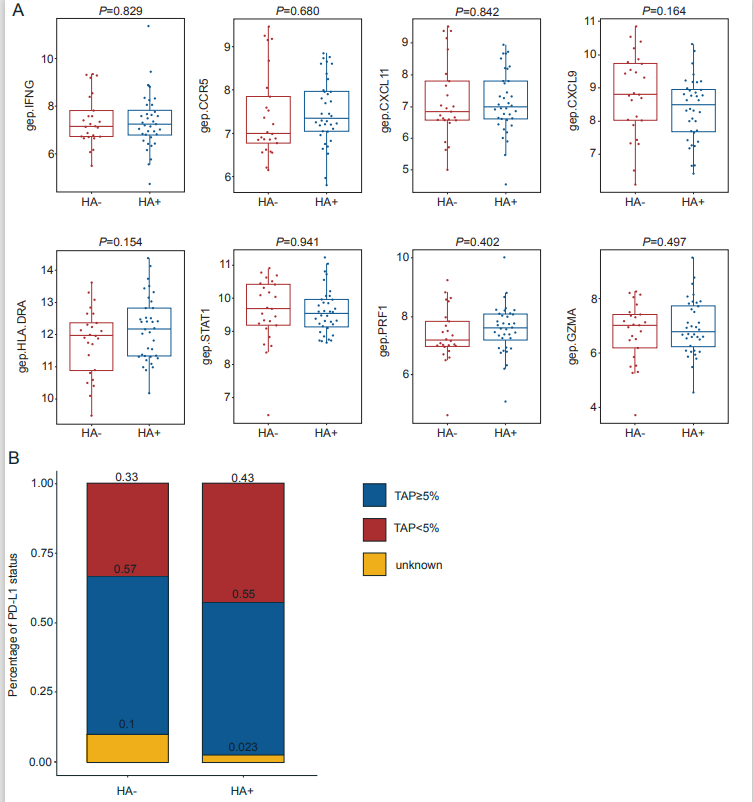


*GEA* gastroesophageal adenocarcinoma, *gep* gene expression profile, *HA* hyperamplification, PD-L1, programmed death-ligand 1, *TAP* Tumor Area Positivity

**Supplementary Fig. 7** The association of joint CD274 status and HA with clinical outcome in the chemotherapy-treated TCGA-STAD independent validation cohort.


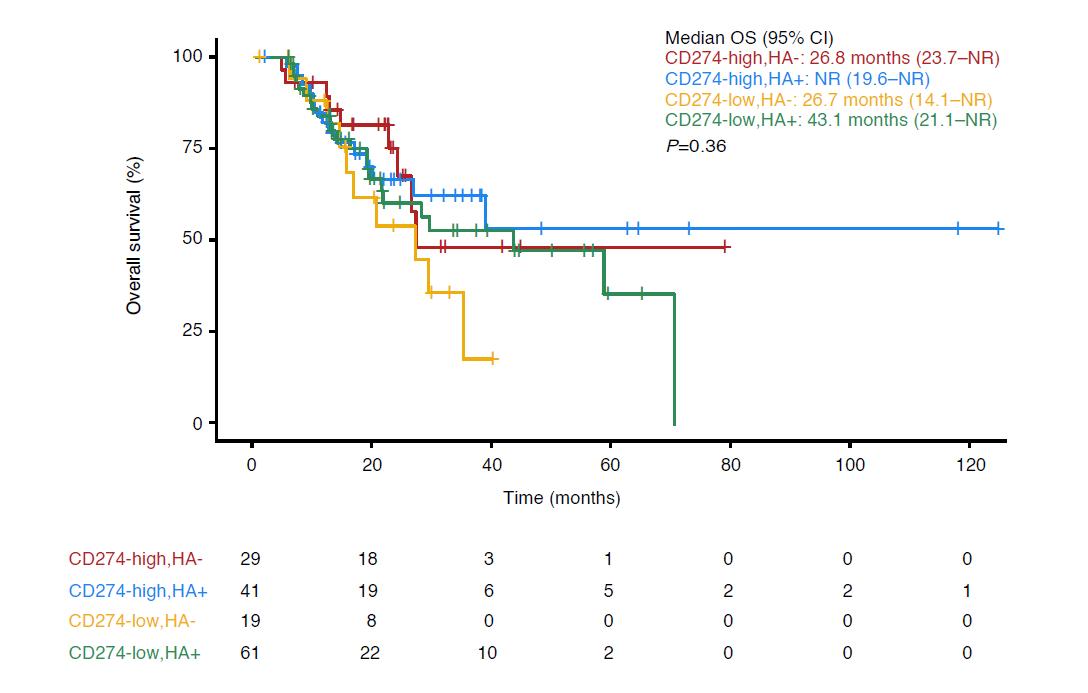


Figure presents a Kaplan-Meier plot for OS according to joint CD274 status and HA.

*CI* confidence interval, *HA* hyperamplification, *NR* not reached, *OS* overall survival, *STAD* stomach adenocarcinoma, *TCGA* The Cancer Genome Atlas

**Supplementary Fig. 8** The association of joint PD-L1 status and HA with CD8+ density by immunohistochemistry in the tumor (**a**) and stroma (**b**) in the tislelizumab-treated GEA cohort.


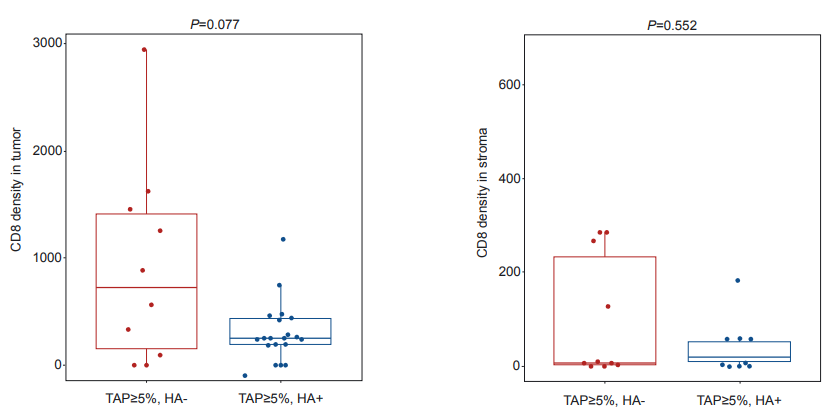


The density of the CD8 immunostained T cells was determined by dividing the number of CD8 T cells (including cells stained with 3+, 2+, and 1+ intensity) by the examined area in mm^2^.

*GEA* gastroesophageal adenocarcinoma, *HA* hyperamplification, *PD-L1* programmed death-ligand 1, *TAP* Tumor Area Positivity

**Supplementary Fig. 9** Volcano plot of differential expressed genes and pathway in TAP≥5%,HA- versus TAP≥5%, HA+ tumors, in the tislelizumab-treated GEA cohort.

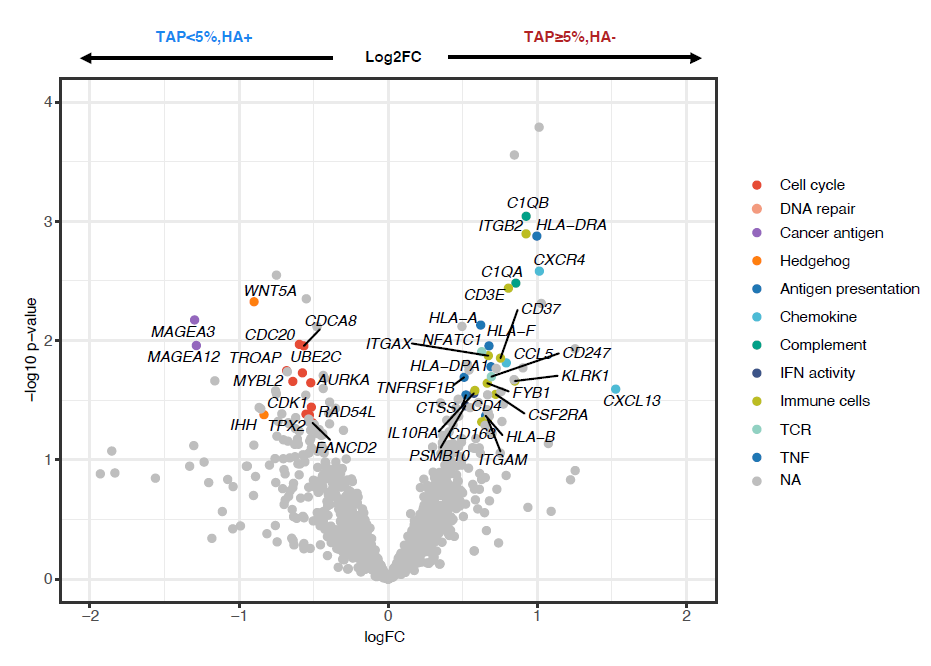


*FC,* fold change, *GEA* gastroesophageal adenocarcinoma, *HA* hyperamplification, *IFN* interferon, *NA* not applicable, *TAP* Tumor Area Positivity, *TCR* T-cell receptor, *TNF* tumor necrosis factor
